# Posterior Component Separation Technique—Original Transversus Abdominis Release (TAR) Technique

**DOI:** 10.3389/jaws.2024.12542

**Published:** 2024-08-21

**Authors:** Jignesh A. Gandhi, Aarsh P. Gajjar, Pravin H. Shinde, Sadashiv Chaudhari

**Affiliations:** ^1^ King Edward Memorial Hospital and Seth Gordhandas Sunderdas Medical College, Mumbai, India; ^2^ Jaslok Hospital, Mumbai, India

**Keywords:** PCST, TAR, abdominal wall reconstruction, Yuri Novitsky, transversus abdominis release

## Abstract

The history of ventral hernia repair techniques has seen substantial evolution, from primary suture repair to the introduction of mesh-based procedures, aiming to reduce recurrence rates. Notable advancements include the anterior and posterior component separation techniques. The Transversus Abdominis Release (TAR) technique, a refinement of the posterior approach, emerged to address challenges associated with complex ventral hernias. The TAR technique facilitates midline reconstruction, allowing large mesh placement while minimizing the need for subcutaneous dissection. Despite its benefits, TAR presents potential complications, emphasizing the necessity for meticulous preoperative assessment and training. The paper reviews the historical progression of hernia repairs, details the TAR technique, highlights indications, perioperative care strategies, surgical steps, postoperative management, technical challenges, and emphasizes the critical role of expertise in achieving successful outcomes in complex abdominal wall reconstruction.

## Introduction

The history of ventral hernia repair techniques has evolved significantly over the years. Initially, primary suture repair was the mainstay of treatment, but this approach was associated with high recurrence rates due to the inherent tension in the repair.

The introduction of mesh-based repairs in the mid-20th century marked a significant advancement in hernia surgery. The use of synthetic mesh to reinforce the repair reduced tension on the suture line and significantly decreased recurrence rates. However, the placement of mesh in contaminated fields was associated with a high risk of infection [1].

Alfonso Roque Albanese pioneered incisions in the oblique muscles, resembling the Ramirez technique, now synonymous with anterior component separation for abdominal wall closure. He expanded his method to include incisions over the oblique minor and posterior rectus sheath, performing component separation hernia repairs in the 1960s to restore the midline without tension in large defects [2]. The Da Silva triple layer repair used the hernia sac to reinforce the rectus sheath, while the peritoneal flap hernia repair reinforced this repair by placing a prosthesis in the Rives-Stoppa plane as described by Andrew de Beaux, to reconstruct the midline without any additional components separation technique [3].

Concurrently, in 1965, French surgeons Dr. Jean Rives and Dr. Rene Stoppa initiated the posterior component separation technique (PCST). The Rives repair initially involved using sublay polyester mesh in the retro-rectus space for inguinal hernia repair. Dr. Stoppa extended this technique to ventral incisional hernias by dissecting the posterior lamina from the rectus muscles and placing polyester mesh in the preperitoneal or retro-rectus space [4]. These techniques gained popularity in the United States through the adaptation by Wantz for unilateral inguinal hernia repair [5]. However, traditional anterior component separation techniques were associated with potential wound complications and recurrence of hernias. This concept was further refined and officially termed “Component Separation” by Ramirez et al. in 1990 [6].

The development of anterior component separation (ACS) involved an incision in the external oblique aponeurosis and dissection between the external and internal oblique muscles. Despite providing greater medial advancement, ACS had higher rates of wound complications and hernia recurrence [7]. Challenges included skin necrosis, technical difficulties in certain patients, and notable wound complications like infection, hematoma, and seroma due to extensive dissection and vessel division [1].

Carbonell et al. [8] described a PCS technique involving lateral division of the Posterior Rectus Sheath (PRS), creating a plane between the Internal Oblique (IO) and Transversus Abdominis (TA) muscle. This technique allowed for myofascial advancement, enhancing midline fascial closure and providing greater space for mesh placement. While postoperative wound complications occurred in 15% of patients, only one patient experienced recurrence. A drawback of this technique was the division of neurovascular bundles, potentially leading to denervation of the RA muscle, which could result in various complications.

In response to these challenges, Yuri Novitsky and colleagues introduced the Transversus Abdominis Release (TAR) technique [9]. TAR is a surgical approach used for complex abdominal wall reconstruction, particularly in the context of large and complex ventral hernias [10, 11]. The development of the PCST, or TAR, was driven by the need for improved methods to manage complex abdominal wall hernias. Traditional ACS techniques had limitations, including the potential for wound complications and recurrence of hernias. The TAR technique was designed to overcome these challenges and improve patient outcomes.

The evolution of PCST from the Rives-Stoppa repair enables the advancement of the RA muscle, facilitating midline closure with the placement of a prosthetic material that extends beyond the retro-rectus space, eliminating the need for subcutaneous tissue dissection. Although not explicitly termed as PCST, the TAR technique reported by Novitsky et al. [9] allowed for bloodless dissection and provided sufficient space for mesh placement by entering the preperitoneal plane. However, this approach exhibited a 24% wound related complications and 4.7% hernia recurrence rate. TAR represents a further refinement of PCS, enabling closure of the PRS and anterior fascia along with the placement of a large mesh in the pre-transversalis pre-peritoneal plane (TAR plane) without disrupting the neurovascular bundles to the RA.

Further modifications of the TAR technique have been described to simplify the surgical procedure and offer additional advantages. For example, a bottom-up TAR technique has been proposed, which allows a simpler dissection beyond the retromuscular space to enable easy approach to perform TAR technique [12].

This emphasizes that the success of the TAR technique requires a thorough understanding of the abdominal wall anatomy, proper training, the adoption of a strict prehabilitation program, and large volumes of experience [10].

## Indications

The TAR technique is primarily indicated for the repair of large and complex ventral hernias, including incisional hernias. Large hernias are defined as hernias with a fascial defect width greater than 10 cm or involving a significant portion of the abdominal wall, typically greater than 25% [13]. It is particularly useful when medial myofascial flap advancement is required [11]. TAR allows for the placement of a large prosthesis in the pre-transversalis pre-peritoneal plane, facilitating midline anatomical reconstruction. TAR is also effective for lateral hernias, particularly those involving the lumbar or flank regions, where traditional approaches may be insufficient for achieving durable repair [14]. It has been shown to be effective in patients with significant comorbidities, such as diabetes and chronic obstructive pulmonary disease [15].

## Perioperative Care and Optimization

Prior to complex abdominal wall reconstruction, a thorough preoperative assessment is recommended. This approach is part of a comprehensive preoperative care strategy that adopts a multidisciplinary team (MDT) approach. This collaborative effort involves specialists from diverse fields, including surgeons, radiologists, anaesthesiologists, nutritionists, and psychologists. Together, they focus on assessing pivotal factors such as abdominal wall musculature, hernia defect dimensions, hernia contents, and loss of domain. The MDT engages in a structured learning process to educate all members with specific learning points pertinent to the procedure. Meticulous preoperative habilitation and comprehensive knowledge about the procedure enhance a smooth post-operative recovery with best surgical outcomes.

Preoperative CT imaging of the abdomen is of great significance, as it offers comprehensive insights into the abdominal wall’s structure, hernia defect dimensions, contents, loss of domain, muscle thickness, retroperitoneal abnormalities, and the previously placed mesh plane. Loss of domain is defined as the ratio of hernia sac volume to the abdominal cavity volume exceeding 20%, indicating that a significant portion of the viscera is contained within the hernia sac. On preoperative CT, surgeons should measure the width and length of the fascial defect and the hernia sac to plan for adequate mesh overlap and ensure complete coverage. Additionally, evaluate muscle thickness, retroperitoneal abnormalities, and previous mesh placement.

Preoperative pulmonary function tests (PFTs) are conducted for all patients. PFTs are recommended for patients with a history of pulmonary disease or significant smoking history to assess baseline lung function and optimize perioperative care [15]. Patient optimization before surgery is crucial for favourable surgical outcomes. Patients with colon as hernia content should receive bowel preparation with polyethylene glycol (PEG) before surgery. Studies suggest that bowel preparation with polyethylene glycol (PEG) can reduce postoperative complications in patients with colon as hernia content, although this is not universally required [16]. There are increased complications in active smokers, poorly controlled diabetic patients, and those who are obese or malnourished [16, 17]. Hence, efforts are made to ensure smoking cessation for at least 4 weeks, strict glycemic control (HbA1c levels < 7 gm%), and weight reduction aiming for a BMI of <30 kg/m^2^ before elective repair. While aiming for a BMI of <30 kg/m^2^ is ideal, a more practical approach is to encourage weight reduction and improve nutritional status as much as possible preoperatively [18]. The team addresses patient-specific considerations like smoking cessation, glycemic control, and weight reduction. These factors play a crucial role in optimizing surgical outcomes and mitigating potential complications linked to smoking, poorly controlled diabetes, obesity, or malnutrition [19]. Progressive corset tightening is supported by anecdotal evidence and expert opinion, but more research is needed to establish its benefits definitively.

The procedure should be carried out under general anaesthesia, with epidural catheter for postoperative regional patient-controlled analgesia (PCA) and prophylactic antibiotics should be given 1 h before incision as per standard institutional protocols. In addition to epidural anesthesia, quadratus lumborum (QL) blocks have shown efficacy in providing postoperative pain relief [20].

Malnutrition is defined by criteria such as unintentional weight loss >10% in the past 6 months, low serum albumin (<3.5 g/dL), and poor dietary intake. Patients should be assessed using tools like the Subjective Global Assessment (SGA) and referred to a nutritionist if identified at risk. A high-protein diet is defined as 1.2–1.5 g/kg body weight/day, and patients should be referred to a nutritionist if their dietary intake is inadequate.

A holistic approach should be adopted for all patients, incorporating anatomical optimization via an abdominal corset designed 2 weeks pre-surgery, physiological optimization using incentive spirometry and progressively tightening the corset to simulate postoperative abdominal pressure increase, nutritional optimization with a high-protein diet, and psychological optimization through preoperative counselling.

## Surgical Technique

The TAR technique has represented a significant milestone in the field of complex abdominal wall reconstruction. This innovative approach involves the development of the retromuscular space, further dividing the posterior lamella of IO muscle to enable division of TA muscle, and getting access into the TAR plane. Gandhi et al. [21] proposed a comprehensive set of procedural principles and guidelines, commonly referred to as the “decalogue,” within the TAR surgical technique. This decalogue encapsulates a refined series of steps and considerations, offering a structured and standardized approach to TAR, thereby enhancing its reproducibility and potentially optimizing patient outcomes.

### Adhesiolysis

A midline laparotomy procedure includes scar excision and entry into the abdomen, guided either by physical examination, selecting an untouched abdominal area, or by reviewing CT scans that display clear pre-peritoneal fat separating the abdominal wall and viscera. Particular attention is paid to avoid visceral injury when the hernial sac is close to the skin. The hernia sac is typically opened in the midline and, as per the case requirements, is often conserved and maintained in continuity with the PRS. Delicate dissection is done with use of cold scissors and appropriate monopolar and bipolar energy devices to perform adhesiolysis from the undersurface of the anterior abdominal wall and the abdominal scar. Inter-bowel adhesiolysis is not mandatory unless patient has presented with episodes of bowel obstructions in the past.

### Placement of the TAR towel

Following this step, a sizable sterile moistened towel is introduced intraperitoneally and carefully positioned, being tucked into the paracolic gutters on the sides, extending inferiorly into the pelvis, and superiorly positioned beneath both domes of the diaphragm. This measure serves the purpose of shielding and safeguarding the viscera throughout the ensuing dissection process.

### Creation of the Rives-Stoppa Plane

**FIGURE 1:**
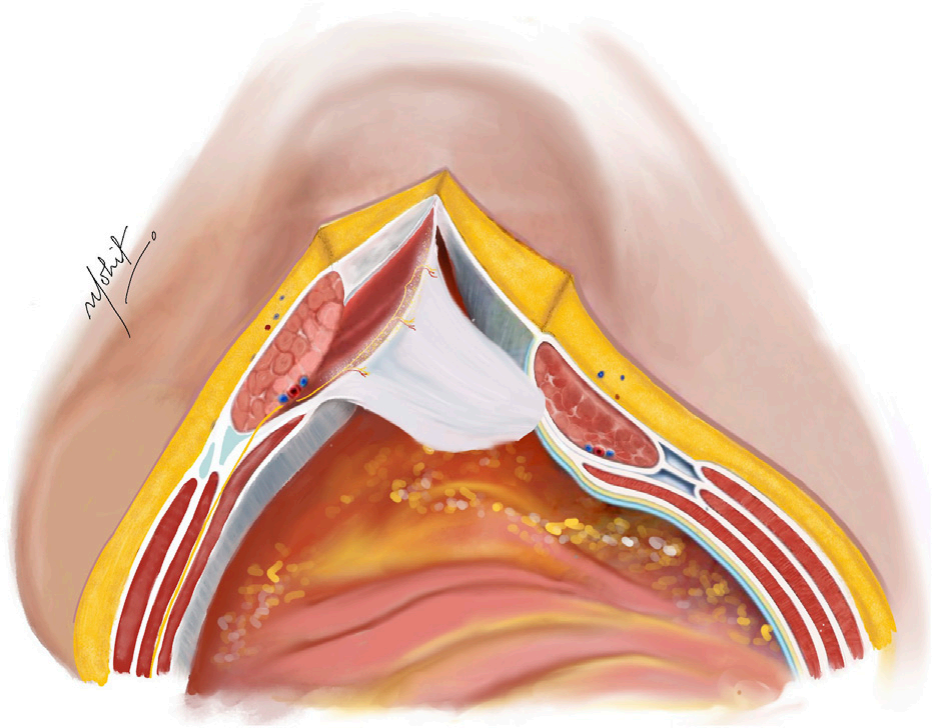
Incision taken on the PRS 5 mm from the medial edge of the rectus abdominis muscle. The hernial sac is kept continuous with the PRS. Illustration by: Dr. Mohit Badgurjar.

An incision is made on the PRS, positioned 5 mm from the midline to enter the Rives-Stoppa plane (Figure 1). This incision is then extended along the entire length in both cranial and caudal directions, revealing the Rives-Stoppa plane. Blunt dissection is employed to carefully develop the plane towards the linea semilunaris, while an assistant retracts the RA muscle superiorly and away from the PRS. In special situations with large sac or wide neck hernias, the medial edge of the RA muscle is identified by palpation or by twitching of its fibres to electrical stimuli. This ensures correct access to the Rives-Stoppa plane. Further, the small perforators from the RA to the PRS are divided with the help of bipolar energy until the lateral most edge of the dissection is reached. Here, the paired neurovascular bundles, representing the linea semilunaris mark the lateral extent of dissection. This can be identified as the “lamppost sign” [22]. Continuing below the arcuate line, dissection progresses to unveil the pre-peritoneal plane, where identification and preservation of the deep inferior epigastric vessels, situated in the pre-transversalis plane along the posterolateral surface of the RA muscle, are crucial. Further inferiorly, dissection extends to reveal the space of Retzius, exposing the pubic symphysis and the Cooper’s ligaments.

In female patients, division of the round ligament can be performed to facilitate good mesh overlap. The division should be done close to the peritoneum to prevent neuropraxia associated with damaging the genital branch of genitofemoral nerve, with the help of energy device or clips.

### Release of TA Muscle

**FIGURE 2:**
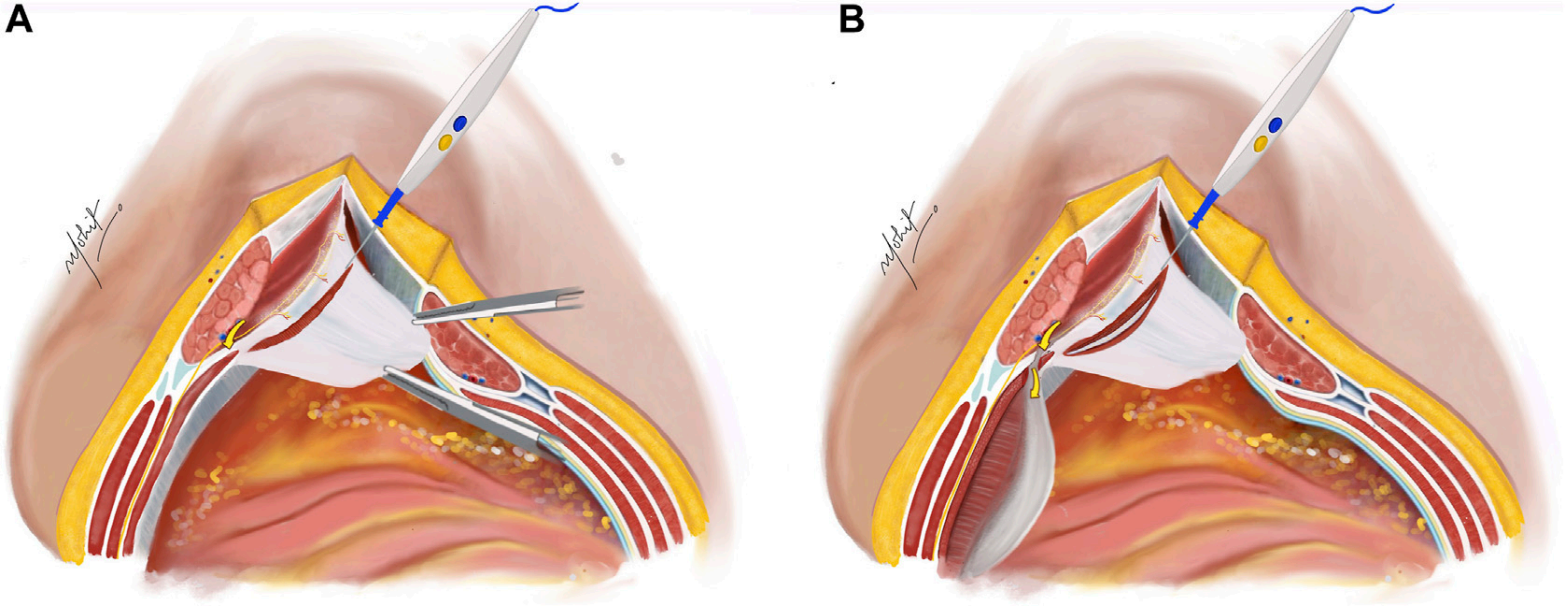
**(A)** The Rives-Stoppa plane is developed. Neurovascular bundles are identified and an incision is taken on the posterior lamella of IO 5 mm medial to the neurovascular bundles to expose the TA muscle. **(B)** Incision taken on the TA muscle to enter the TAR plane. Illustration by: Dr. Mohit Badgurjar.

The subsequent step involves a 5 mm incision on the posterior lamella of the IO aponeurosis, positioned medially to the neurovascular bundles to expose the underlying TA muscle (Figure 2). This incision is typically performed in the middle third of the plane where the TA fibres are muscular, compared to the lower third where the muscle is more aponeurotic. Utilizing the “bottom-up” approach in select cases becomes feasible due to this fat presence, facilitating the establishment of TAR plane. The TA muscle is raised using Lahey’s forceps, and small bites of short bursts of monopolar and/or bipolar energy is used to divide the muscle fibres. This approach continues in the cranial as well as caudal aspects of the plane.

### Creation of the TAR Plane

**FIGURE 3:**
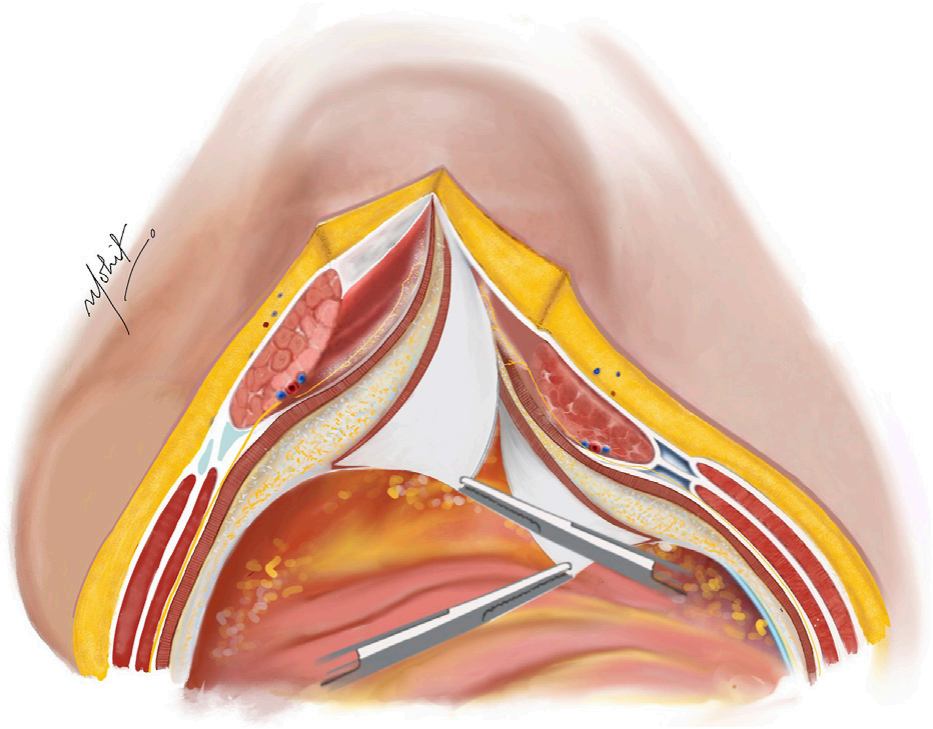
The TA muscle lifted up and away from the fascia transversalis. The TAR plane is developed laterally until the psoas muscle. Illustration by: Dr. Mohit Badgurjar.

Using novel right-angled retractor, the assistant elevates the abdominal wall upward, while the surgeon’s left hand applies a counter pressure over the TAR plane pushing the TA muscles upwards approaching the lateral extraperitoneal space (Figure 3). An additional mop is positioned to provide added safeguarding over the fascia transversalis/peritoneum. Employing a peanut dissector, the TA muscle is lifted superiorly and separated from the transversalis fascia/peritoneum. Lateral dissection is extended toward the psoas muscle, avoiding exposure of ureters. The dissection extends caudally up to the space of Retzius in the midline, and laterally the space of Bogros. In select cases of M1 and M2 hernias, the dissection extends cranially in the pre-diaphragmatic space up to the central tendon of diaphragm. Any peritoneal button holes are sutured using absorbable material. For larger defects, omentum is used for reinforcement.

### TAR on the Other Side

A similar process is carried out on the opposite side by dividing the posterior lamella of IO, further dividing the TA muscle and creating a TAR plane. This enables substantial medialisation of the RA muscles (8–12 cm on each side) [23].

### Closure of the PRS

**FIGURE 4:**
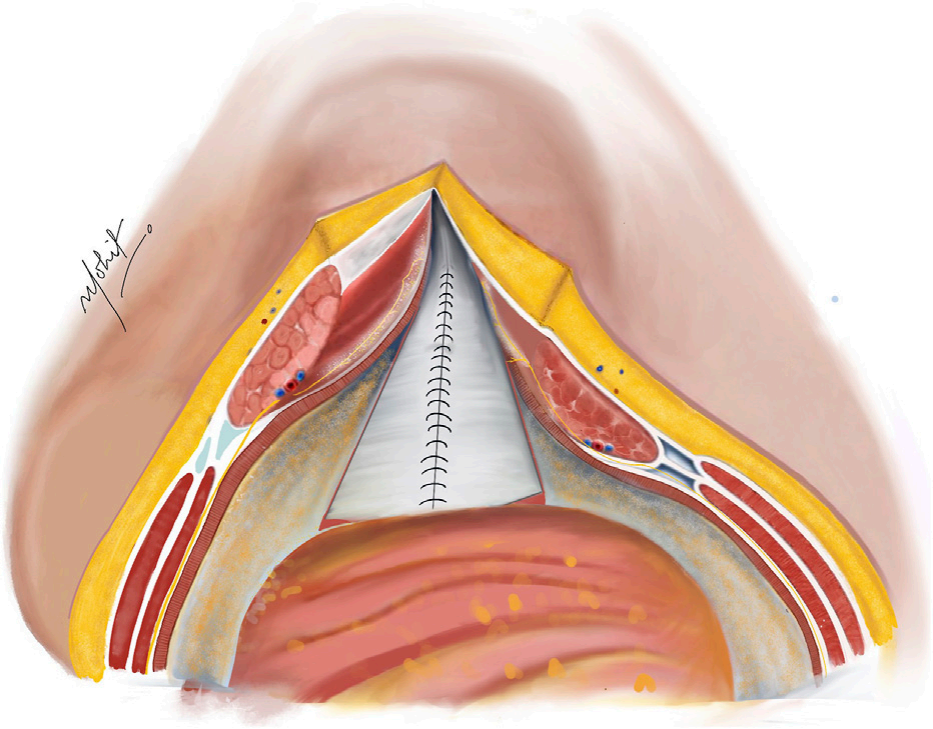
Complete closure of the PRS done with absorbable sutures after completion of bilateral TAR; in cases of incomplete approximation of the posterior rectus sheath (PRS), hernial sac kept continuous with the PRS is used to achieve closure. Illustration by: Dr. Mohit Badgurjar.

The PRS is approximated in the midline after removal of the TAR towel with closely spaced 5–8 mm suture bites using 1-0 absorbable polyglactin suture with a cranial and a caudal approach completing it in the middle (Figure 4). In difficult cases of PRS approximation, the use of polyglactin mesh, biosynthetic mesh and omentum as a patch is recommended. It is important to monitor the peak/plateau pressure during and at the end of PRS closure. Following this, a transversus abdominis plane (TAP) block is administered by injecting liposomal bupivacaine into the intramuscular plane between the IO and TA muscles using an 18-gauge needle under direct visualization [20].

### Placement of the Mesh

**FIGURE 5:**
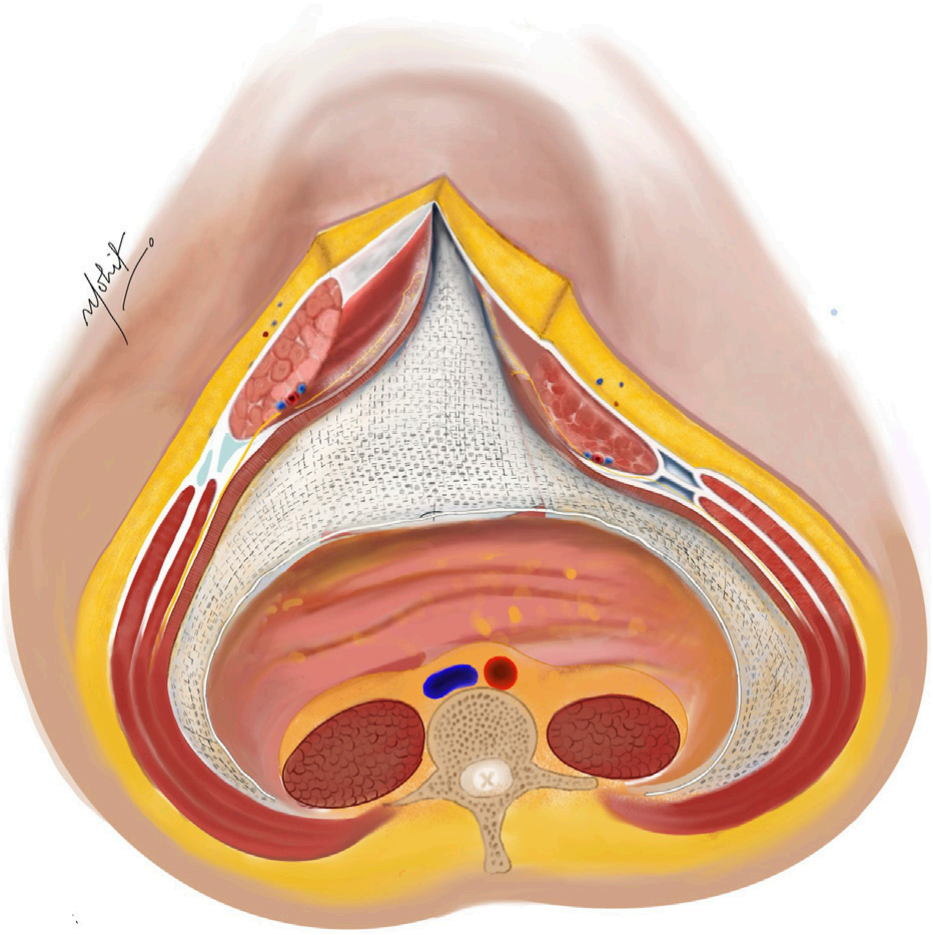
A medium weight polypropylene mesh placed in the TAR plane and fixed laterally under physiological tension. Linea alba reconstructed ventrally to the mesh using non-absorbable sutures. Illustration by: Dr. Mohit Badgurjar.

**FIGURE 6:**
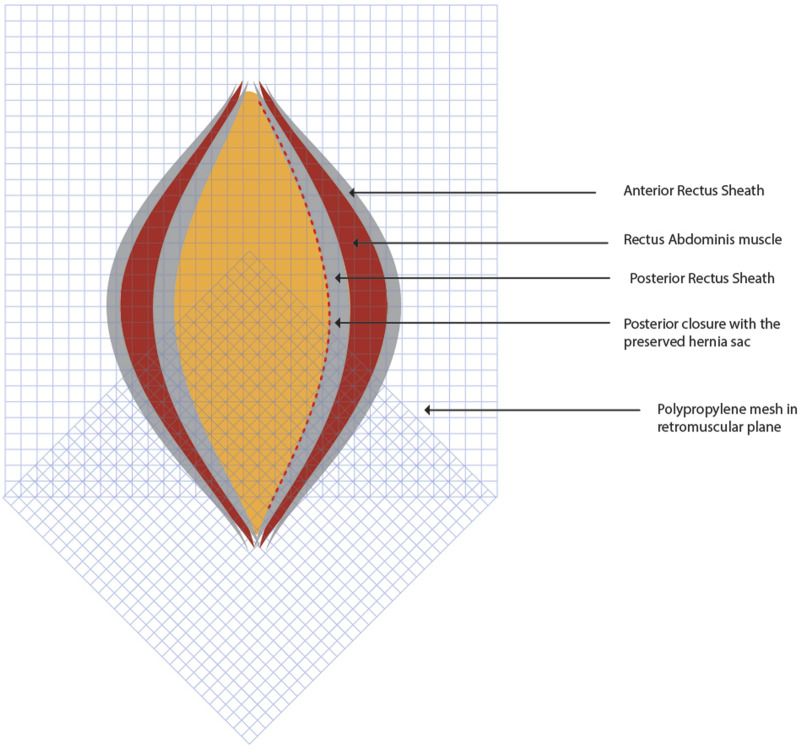
Two 30 cm × 30 cm medium weight polypropylene meshes are placed in the TAR plane in home plate configuration. Figure inspired from Gandhi et al [21].

Two 30 cm × 30 cm medium weight polypropylene meshes (MWPP) arranged in a home plate configuration are placed in the TAR plane. Using two meshes in a home plate configuration provides better coverage and ensures overlap, especially in very large defects. However, a single large mesh may suffice for smaller defects. Optional fixation of the mesh can be done with transfascial fixation under physiological tension to prevent folding of the mesh laterally (Figures 5, 6). Two closed suction drains are placed in the TAR plane to mitigate seroma formation.

### Closure of the Anterior Rectus Sheath (ARS)

The ARS is then approximated with 1-0 interrupted nylon sutures with a cranial and a caudal approach to reconstruct the midline in front of the mesh (Figure 5). Interrupted nylon sutures provide greater tensile strength and allow for adjustments in tension during closure. However, running slowly absorbable sutures are also effective and commonly used. Monitoring of the peak/plateau pressure is pertinent during this step to prevent future compartment syndrome [24]. Surgeons and anaesthesiologists should monitor for signs of abdominal compartment syndrome, such as elevated peak inspiratory pressures (>20 cm H_2_O) and decreased urine output. As a mitigation strategy for the difficult closure of ARS, a heavy weight polypropylene (HWPP) mesh can be used to bridge the midline. When using heavyweight polypropylene mesh to bridge the midline, it should be secured with non-absorbable sutures to the fascia on either side, ensuring no tension on the mesh [11].

### Subcutaneous Tissue and Skin Closure

A flat drain is inserted into the subcutaneous plane to avoid seroma. The subcutaneous tissue is stitched using a 2-0 polyglactin absorbable material, and the skin is closed with staples or interrupted sutures. An accurately fitted abdominal corset is applied immediately.

## Discussion

### Pros and Cons of TAR Technique

The Transversus Abdominis Release (TAR) technique facilitates substantial medial advancement of the rectus abdominis muscles, typically achieving 8–12 cm on each side, in contrast to the 5–7 cm achieved with anterior component separation [7, 23]. This method is associated with reduced wound complications due to the minimized need for subcutaneous dissection [15]. Additionally, TAR enhances mesh integration and decreases infection risk owing to the better vascularization of the placement site [10]. The technique is particularly effective for patients with significant comorbidities such as obesity and diabetes [15].

However, the TAR technique demands a high level of expertise and an in-depth understanding of abdominal wall anatomy, limiting its practice to highly skilled surgeons [16]. The steep learning curve associated with TAR necessitates extensive training and practice, which can impede its widespread adoption [25]. Despite its advantages, TAR can result in complications such as seroma formation, wound infection, and recurrence, although these rates are generally lower compared to other techniques [11]. Furthermore, the procedure often requires longer operative times and specialized equipment, making it resource-intensive [10].

It is crucial for TAR to be performed by surgeons with specialized training and extensive experience in hernia repair to ensure optimal outcomes and minimize potential complications [16].

### Technical Challenges and Mitigation Strategies

The original TAR technique, as described by Yuri Novitsky, has been associated with several potential complications and limitations. Surgical site events, including infections, have been reported in up to 18.7% of cases, with surgical site infections specifically occurring in 9.1% of cases. In some instances, these complications have necessitated mesh debridement, although complete mesh explantation has been reported as rare. Despite the technique’s overall effectiveness, recurrences have been reported in up to 3.7% of patients with at least 1-year follow-up [11].

Furthermore, the TAR technique requires a thorough understanding of the abdominal wall anatomy and proper training, which may limit its widespread adoption [10]. One significant concern lies in the delicate dissection required to access the TAR plane, avoiding neurovascular bundles, and adapting to anatomical landmarks. Potential complications include “mickey mouse” hernia due to injury to the semilunar line, intraparietal hernias from improper closure of the posterior rectus sheath, and consequences of holes in the posterior layer. Meticulous attention to surgical technique can mitigate these risks.

Surgeons embarking on the implementation of the PCSTAR techniques should undergo specialized training in designated centres of excellence. This training encompasses immersive experiences in cadaver workshops, providing hands-on practice and refinement of the intricate dissection techniques required for accessing the TAR plane [25]. Additionally, surgeons have access to a wealth of educational resources, including peer-reviewed videos and articles, which offer valuable insights and best practices for executing PCSTAR with precision and proficiency. This comprehensive training regimen equips surgeons with the necessary skills and knowledge to navigate the technical challenges associated with PCSTAR, emphasizing the critical role of expertise and meticulous surgical technique in achieving successful outcomes in complex abdominal wall reconstruction.

### Postoperative Care and Outcomes

Postoperative care prioritizes minimizing the use of narcotics while focusing on measures like adequate regional PCA, prophylaxis against deep vein thrombosis, utilization of abdominal corsets, prescribed bed rest, chest physiotherapy and adherence to the Enhanced Recovery After Surgery (ERAS) protocol. Drain removal is contingent on output, and examining the midline wound is delayed as a standard procedure. Long-term outcomes are evaluated through regular follow-up appointments and CT scans.

To reduce reliance on narcotics, the TAP block is utilized for effective pain relief in most patients. Additionally, acetaminophen is administered to provide supplementary pain relief. Initiation of deep vein thrombosis prophylaxis aligns with current recommendations [26]. Patients are advised to continue wearing the abdominal corset, start early mobilization to reduce the risk of thromboembolism and improve recovery, bed rest should be minimized, and follow an ERAS protocol with a shift to clear liquids on the first day after surgery [27, 28]. Transition to a soft diet occurs after the complete resolution of postoperative ileus. Drains are removed when daily output falls below 20 mL. Inspection of the midline wound is deliberately postponed, unless dressing soakage necessitates earlier attention. Incisional wound vacs can be beneficial in reducing infection rates and improving healing. Dressings should be changed based on clinical assessment.

Post discharge, follow-up should be scheduled at 2 weeks, 1 month, 3 months, 6 months, and annually thereafter, with additional visits as needed based on clinical findings. Routine CT scans are recommended at 6 months and 1 year postoperatively to monitor for hernia recurrence and other complications.

## Conclusion

In conclusion, the evolution of ventral hernia repair techniques has progressed significantly over time, transitioning from primary anatomical repair to components separation technique and subsequently introducing complex surgical approaches such as the TAR technique. TAR, as a refined component of the posterior component separation technique, has demonstrated its efficacy in managing large and complex ventral hernias, achieving substantial medialization of rectus abdominis muscles and facilitating midline closure while providing a space for large mesh placement. However, the successful application of TAR demands a thorough understanding of abdominal wall anatomy, specialized training, and adherence to strict prehabilitation protocols.
